# Supplementary material for: Empirical methods for controlling false positives and estimating confidence in ChIP-Seq peaks
Source: BMC Bioinformatics. 2008 Dec 5;9:523. doi: 10.1186/1471-2105-9-523 (PMC2628906; doi:10.1186/1471-2105-9-523)
Supplement: Additional File 1 — A variety of html documents from the USeq web site detailing the available applications, their best usage, output file type descriptions, command line menus, etc. [file 1471-2105-9-523-S1.zip › USeqUserGuides/JavaDoc.zip!/JavaDoc/trans/qc/CoordinateExtractor1lq.html]

CoordinateExtractor1lq


|  |  |  |
| --- | --- | --- |
| **Overview** \| **Package** \| **Class** \| **Use** \| **Tree** \| **Deprecated** \| **Index** \| **Help** | |  |
| **PREV CLASS**   **NEXT CLASS** | **FRAMES**    **NO FRAMES**     **All Classes** |  |
| SUMMARY: NESTED \| FIELD \| CONSTR \| METHOD | DETAIL: FIELD \| CONSTR \| METHOD |  |


---


## trans.qc Class CoordinateExtractor1lq

```
java.lang.Object
  trans.qc.CoordinateExtractor1lq
```

---

``` public class CoordinateExtractor1lq extends java.lang.Object ```

Extracts control an pm lines from a text 1lq file.
A serialized int[][][] for the xy coordinates is saved.

---

**Constructor Summary**

`CoordinateExtractor1lq(java.lang.String[] args)`


| **Method Summary** | |
| --- | --- |
| `static int[]` | `countNumberIntensities(int[][][] coordinates)`             Calculate number of noSynth, dim, bright controls, and pm probes. |
| `int[][][]` | `extract1lqFile(java.io.File onelqFile, java.io.File outputFile)`             Fetches coordinates for DESTYPEs of 0, 3, -3, 6, or -6 (Control) and PM -111, 111. |
| `void` | `extract1lqFiles()`             Extracts control oligo information from 1lq files. |
| `static void` | `main(java.lang.String[] args)` |
| `static float[][]` | `makeVirtualCel(int[][] xyCoor, int numRows)`             Make a virtual cel file loading xyCoors with an intensity of 100. |
| `static void` | `printDocs()` |
| `void` | `processArgs(java.lang.String[] args)`             This method will process each argument and assign any new varibles |

**Methods inherited from class java.lang.Object**

`equals, getClass, hashCode, notify, notifyAll, toString, wait, wait, wait`

**Constructor Detail**

### CoordinateExtractor1lq

```
public CoordinateExtractor1lq(java.lang.String[] args)
```


**Method Detail**

### extract1lqFile

```
public int[][][] extract1lqFile(java.io.File onelqFile,
                                java.io.File outputFile)
```

:   Fetches coordinates for DESTYPEs of 0, 3, -3, 6, or -6 (Control) and PM -111, 111.
    Lumps sense and antisense together -6/6, -3/-3, -111/111
    int[probe index][x,y] order noSynth, dim, bright, and pm.
    Returns null if a problem is encountered.

---


### extract1lqFiles

```
public void extract1lqFiles()
```

:   Extracts control oligo information from 1lq files.

---


### makeVirtualCel

```
public static float[][] makeVirtualCel(int[][] xyCoor,
                                       int numRows)
```

:   Make a virtual cel file loading xyCoors with an intensity of 100.

---


### countNumberIntensities

```
public static int[] countNumberIntensities(int[][][] coordinates)
```

:   Calculate number of noSynth, dim, bright controls, and pm probes.

---


### main

```
public static void main(java.lang.String[] args)
```

---


### processArgs

```
public void processArgs(java.lang.String[] args)
```

:   This method will process each argument and assign any new varibles

---


### printDocs

```
public static void printDocs()
```


---


|  |  |  |
| --- | --- | --- |
| **Overview** \| **Package** \| **Class** \| **Use** \| **Tree** \| **Deprecated** \| **Index** \| **Help** | |  |
| **PREV CLASS**   **NEXT CLASS** | **FRAMES**    **NO FRAMES**     **All Classes** |  |
| SUMMARY: NESTED \| FIELD \| CONSTR \| METHOD | DETAIL: FIELD \| CONSTR \| METHOD |  |


---
